# Supplementary material for: Super‐Resolution Imaging of Nanoscale Inhomogeneities in hBN‐Covered and Encapsulated Few‐Layer Graphene
Source: Adv Sci (Weinh). 2025 Feb 14;12(14):2409039. doi: 10.1002/advs.202409039 (PMC11984919; doi:10.1002/advs.202409039)
Supplement: Supplementary file 1 — Supporting Information [file ADVS-12-2409039-s001.docx]

**Supplementary Information:**

**Super-Resolution Imaging of Nanoscale Inhomogeneities in hBN-Covered and Encapsulated Few-Layer Graphene**

Author(s) and Corresponding Author(s)*:

Lina Jäckering*^,1,2^, Konstantin G. Wirth^1,2^, Lukas Conrads^1,2^, Jonas B. Profe^3,4^, Alexander Rothstein^5,6^, Hristiyana Kyoseva^5^, Kenji Watanabe^7^, Takashi Taniguchi^8^, Dante M. Kennes^4,9^, Christoph Stampfer^2,5,6^, Lutz Waldecker^2,5^, Thomas Taubner^1,2^

[jaeckering@physik.rwth-aachen.de](mailto:jaeckering@physik.rwth-aachen.de)

**Affiliations**

^1^1st Institute of Physics (IA), RWTH Aachen University, 52074 Aachen, Germany

^2^Juelich-Aachen Research Alliance (JARA-FIT), 52425 Juelich, Germany

^3^Institute for Theoretical Physics, Goethe University Frankfurt, 60438 Frankfurt a.M., Germany

^4^Institute for Theory of Statistical Physics, RWTH Aachen University and JARA Fundamentals of Future Information Technology, 52062 Aachen, Germany

^5^2nd Institute of Physics, RWTH Aachen University, 52074 Aachen, Germany

^6^Peter Grünberg Institut (PGI-9), Forschungszentrum Jülich, 52425 Jülich, Germany

^7^Research Center for Electronic and Optical Materials, National Institute for Materials Science, 1-1 Namiki, Tsukuba 305-0044, Japan

^8^International Center for Materials Nanoarchitectonics, National Institute for Materials Science, 1-1 Namiki, Tsukuba 305-0044, Japan

^9^Center for Free Electron Laser Science, Max Planck Institute for the Structure and Dynamics of Matter, 22761 Hamburg, Germany

**This PDF file includes:**

**Supplementary Note 1: Assignment of the stacking orders in the TLG flake**

**Supplementary Note 2: Stacking-dependent polaritons in hBN-covered TLG**

**Supplementary Note 1: Assignment of the stacking orders in the TLG flake**


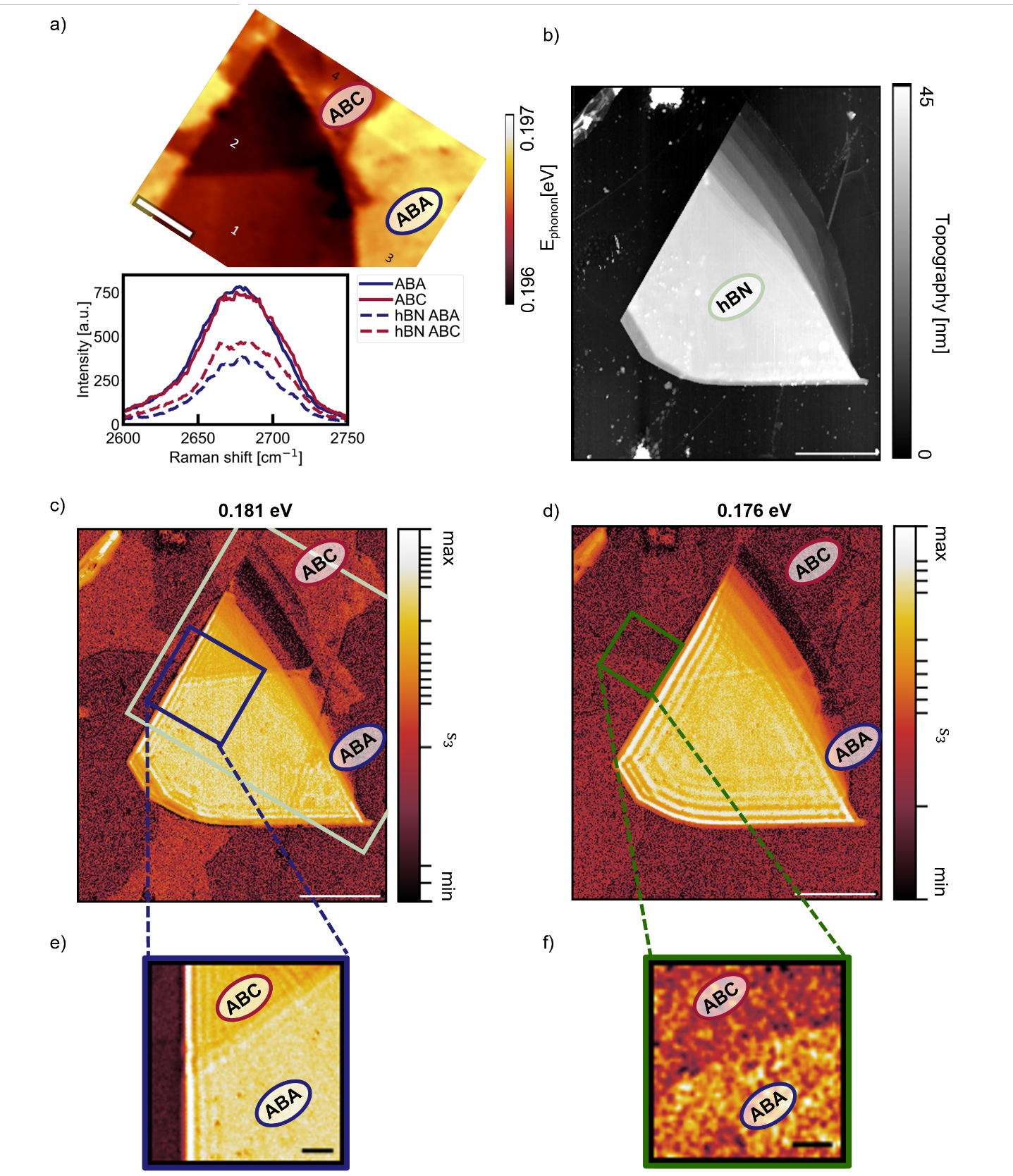


**Figure S1:** **Assignment of the stacking orders in the TLG flake.** a) Raman G-peak map (top) of the TLG flake partly covered with an hBN flake and Raman 2D-peak spectra for the four areas with distinct G-peak positions (bottom). The scale bar corresponds to 4 µm. b) AFM topography image of the same flake as in a) acquired simultaneously with the s-SNOM amplitude image in c). c) and d) s-SNOM amplitude images of the same hBN TLG heterostructure as in a) and b) at 0.181 and 0.176 eV, respectively. The light green rectangle in c) indicates the area of the Raman G-peak map in a). The blue (in c)) and green (in d)) rectangles mark the areas of the amplitude images shown in the main text in Figure 1g at an hBN edge and 1d of the uncovered TLG, also shown in e) and f), respectively. The scale bars in b)-d) correspond to 5 µm and in e) and f) to 1µm.

We support the assignment of the stacking orders in the investigated TLG flake in Figure 1 in the main text with Raman spectroscopy^[1,2]^. A map of the integrated G-peak intensity from 0.196 to 0.197 eV is shown in Figure S1a (top). The darker areas (labeled ‘1’ and ‘2’) correspond to TLG covered with hBN, while the lighter parts are uncovered TLG. In both, covered and uncovered TLG, we observe distinct domains. Raman spectra around the 2D peak (shown in the bottom) allow to assign these different areas to ABA and ABC stacking orders as the 2D peak shows a sharp side peak and an enhanced shoulder for ABC stacked TLG. ^[1,2]^

In the AFM topography image (Figure S1b) we observe a triangular-shaped area of high topography with a step-like height profile in the upper right. This triangular-shaped area corresponds to the hBN flake covering the TLG. The s-SNOM amplitude images of the same sample taken at 0.181 and 0.176 eV (Figure S1c and S1d) reveal a high s-SNOM amplitude in the area of the hBN flake due to hBN’s high permittivity at the lower bound of the upper reststrahlenband. At the edges of the hBN flake, we observe bright fringes arising from the interference of polaritons in the heterostructure as discussed in Figure 2 in the main text.

At 0.181 eV (c.f. Figure S1c) the heterostructure shows a lower s-SNOM amplitude in the upper part of the hBN-covered TLG. Supported by the Raman G-peak map showing a lower G-peak energy in this area, we assign the upper part of the hBN-covered TLG to the hBN-covered ABC TLG. The rest of the hBN flake covers ABA TLG. The area of uncovered TLG shows areas of two distinct s-SNOM amplitudes. These areas agree with the areas that show distinct G-peak energies on the right of the Raman map in Figure S1a. Thus, we assign the uncovered areas with lower s-SNOM amplitude to ABA TLG and those with higher s-SNOM amplitude to ABC TLG.

At 0.176 eV (c.f. Figure S1d) we do not observe areas of different s-SNOM amplitudes in the area of the hBN-flake. In the uncovered TLG area we observe the same TLG domains as at 0.181 eV, but with the ABA TLG showing a higher s-SNOM amplitude and the ABC TLG showing a lower s-SNOM amplitude. The inversed contrast arises from the energy-dependent conductivities of the stacking orders that also lead to an energy-dependent s-SNOM amplitude contrast between the stacking orders.

The blue and green rectangles in Figure S1c and S1d, also shown in Figure S1e and S1f, respectively mark the areas of the s-SNOM amplitude images shown in Figure 1g and 1d in the

main text, respectively.

**Supplementary Note 2: Stacking-dependent polaritons in hBN-covered TLG**


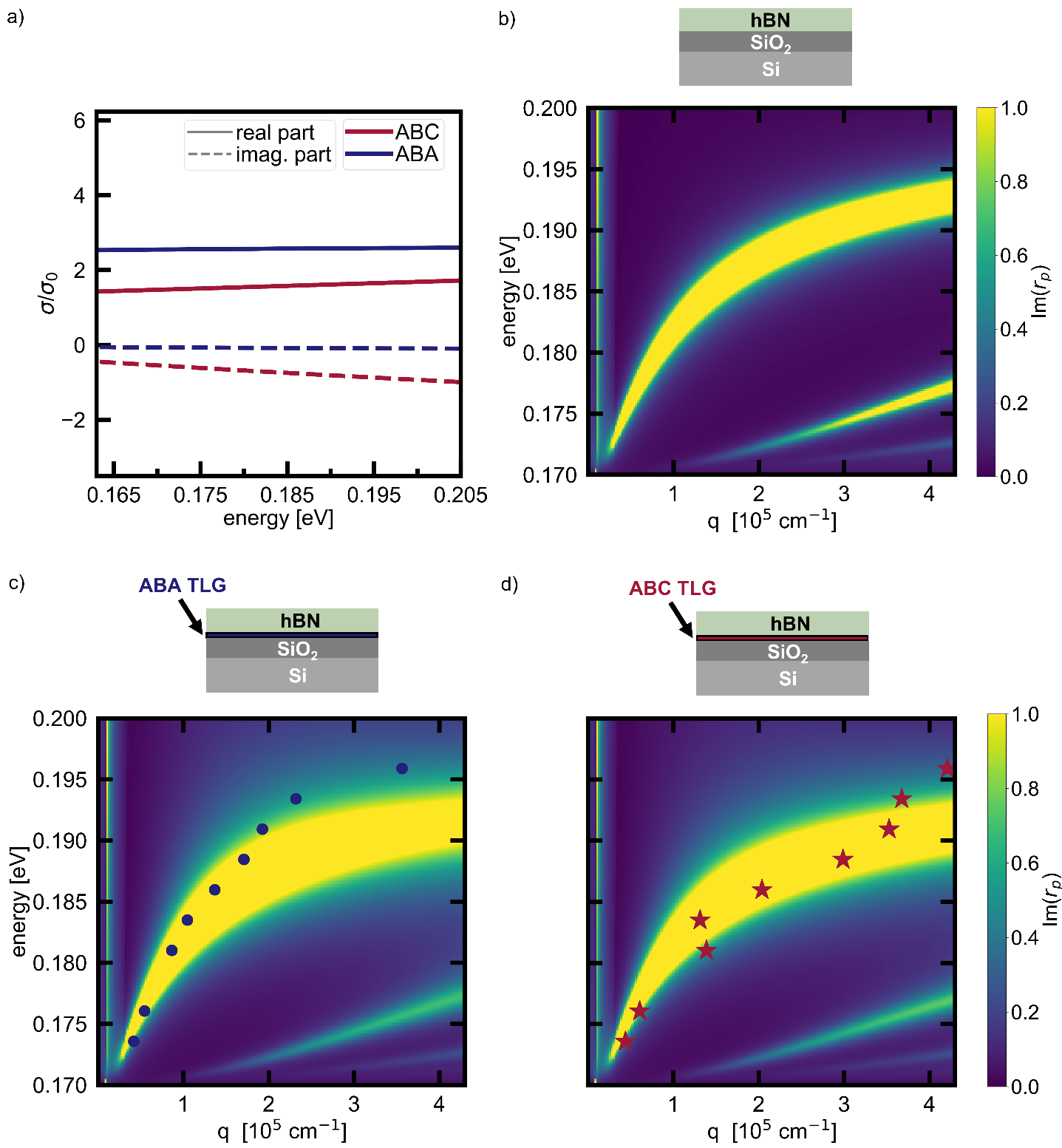


**Figure S2:** **Hybridization of the plasmon polaritons in TLG and the hyperbolic phonon polaritons in hBN leading to the stacking-dependent dispersion.** a) Real (solid line) and imaginary (dashed line) part of the optical conductivities of ABA (blue) and ABC (red) TLG in the investigated energy regime. b) Modeled dispersion of the hyperbolic phonon polaritons in a 33 nm thick hBN slab on the SiO_2_-Si. c) and d) Comparison of the experimentally obtained (blue dots in c) and red stars in d)) and modeled dispersion of the hyperbolic phonon plasmon polaritons for the heterostructure with ABA TLG and ABC TLG, respectively.

Figure S2a shows a zoom-in of the optical conductivities of ABA and ABC TLG shown in Figure 1a and 1b in the investigated energy regime. In this energy regime ABC TLG’s optical conductivity shows a higher magnitude in the imaginary part and a lower real part than ABA TLG’s conductivity.

The modeled dispersion relation (color plot of the imaginary part of the reflection coefficient) is calculated for an hBN slab on the SiO_2_-Si substrate (Figure S2b) and the two heterostructures (hBN ABA TLG in Figure S2c and hBN ABC TLG in Figure S2d) using the transfer matrix method.^[3]^ The dispersion is calculated for the sample stacks sketched at the top of S2b-S2d, respectively. The SiO_2_ is assumed to have a thickness of 90 nm. The hBN thickness is 33 nm. The permittivity of hBN is shown in Figure 1b and the optical conductivities of the TLG stacking orders are in Figures 1d and 1e.

For a pure hBN slab (Figure S2b) we observe a pronounced principal phonon polariton branch and two higher-order phonon polariton modes at high q-vectors and low energies. The higher-order modes arise from the waveguide-like propagation of the hBN phonon polaritons. In the heterostructures with hBN on top of ABA and ABC TLG, the modes are broadened and shifted to slightly lower energies. Comparing the two heterostructures we observe that the principal branch of the ABC heterostructure is shifted to slightly higher wavevectors compared to that of the ABA heterostructure. In the heterostructure of hBN on top of TLG, the phonon polaritons couple to the surface plasmon polaritons in TLG. Therefore, we observe hybridized modes in the dispersion of the heterostructure. Since the two stacking orders of TLG differ in their optical conductivities, the TLG surface plasmon polaritons are stacking-dependent^[4]^ and we observe differently hybridized modes in the heterostructures with hBN on top of ABA and on top of ABC TLG.

For both heterostructures, the experimental data agree with the principal mode in the modeled dispersion. The experimental dispersion of the ABA heterostructure lies at slightly higher energies than the modeled dispersion for energies above 0.19 eV whereas the experimental dispersion of the ABC heterostructure agrees with the modeled dispersion up to an energy of 0.193 eV.

**References**

[1] C. Cong, T. Yu, K. Sato, J. Shang, R. Saito, G. F. Dresselhaus, M. S. Dresselhaus, *ACS nano* **2011**, *5*, 8760.

[2] C. H. Lui, Z. Li, Z. Chen, P. V. Klimov, L. E. Brus, T. F. Heinz, *Nano letters* **2011**, *11*, 164.

[3] T. Zhan, X. Shi, Y. Dai, X. Liu, J. Zi, *Journal of Physics: Condensed Matter* **2013**, *25*, 215301.

[4] Y. Luan, J. Qian, M. Kim, K.-M. Ho, Y. Shi, Y. Li, C.-Z. Wang, M. C. Tringides, Z. Fei, *Phys. Rev. Applied* **2022**, *18, 2*.
